# End-of-Life Care in Patients with Cancer 16–24 Years of Age

**DOI:** 10.1007/s11912-021-01173-0

**Published:** 2022-01-25

**Authors:** Natacha D. Emerson, Krista Tabuenca, Brenda Bursch

**Affiliations:** 1 Department of Psychiatry, David Geffen School of Medicine, UCLA, Los Angeles, CA USA; 2 Department of Pediatrics, David Geffen School of Medicine, UCLA, Los Angeles, CA USA

**Keywords:** End-of-life, Oncology, Adolescents, Young adults, Palliative care

## Abstract

**Purpose of Review:**

To present new findings in order to aid in the provision of high-quality symptom management and psychosocial care for adolescents and young adults with advanced cancer at the end of life.

**Recent Findings:**

Behavioral health providers support patients by teaching them symptom control skills, building legacies, and making meaning of their lives. Integration of cultural values is essential for comprehensive assessment and decision-making. Effective management of physiological symptoms and psychological distress begins with accurate communication about prognosis and goals of care that focus on patient preferences and priorities. Oncology teams promote quality of life and the successful management of fatigue, pain, decreased mobility, poor appetite, and dyspnea with the early inclusion of palliative care.

**Summary:**

While provision of end-of-life care in a young person with cancer presents challenges, multidisciplinary teams can effectively accompany patients in this journey by prioritizing patient and family preferences to promote quality of life.

## Introduction

Although rare, cancer is the leading cause of death due to disease in persons 16 to 24 years of age. It is also the fourth leading cause of death in this age group due to any cause, after accidents, suicide, and homicide [1]. In this age group, lymphomas and thyroid cancer are the most common cancers [2].

Ethnic and income survival disparities among adolescents and young adults with cancer have increased over time. Black patients with cancer in this age range are at highest risk for death, followed by Asian/Pacific Islander, then LatinX White patients [3]. Additionally, lower income adolescents and young adults with cancer are at increased risk for death compared to higher income patients [3]. The type of cancer does not account for these differences, highlighting the importance of improving access to cancer education, screening, and quality health care.

Ideal end-of-life care for youth 16–24 years of age utilizes the best available evidence delivered with cultural sensitivity to meet the unique challenges faced by this age group and their families.

## Defining End-of Life Care

*Advanced cancer* refers to cancers that cannot be cured, meaning that treatment will not result in the cancer remitting without future recurrence [4]. However, some types of advanced cancer can be treated to slow disease progression and to address bothersome symptoms. It is possible for life to be prolonged for weeks to years with reasonable quality of life (QOL) despite the cancer persisting. Some individuals learn they have advanced cancer at the time of initial diagnosis; for others, cancer may not be deemed advanced until years later. Metastatic cancer, meaning cancer that has spread away from its site of origin to other parts of the body, may or may not be curable based on the type of cancer [5]. End-of-life care refers to the physical, emotional, social, and spiritual support provided to an individual during the days, weeks, or months leading up to death. For some, this means that aversive treatments are discontinued [6] and the focus shifts to palliation. In this case, the primary goal should be to help the individual meet their own goals, such as optimizing comfort and/or functioning.

## Case Illustration

Manuel is an 18-year-old LatinX, cisgender male with osteosarcoma of the humerus. Shortly after high school graduation, he reports sudden onset right shoulder pain that turns out to be cancer. Manuel is an avid baseball player with dreams of playing in the major league. Both his oncologist and surgeon recommend a right arm amputation as the treatment with the best prognostic indicators. Crushed about what this means for his athletic future, Manuel refuses and opts for local resection and inpatient chemotherapy. Only 4 months later, Manuel is told his cancer has recurred in his arm and undergoes an amputation. Two months later, scans reveal evidence of pulmonary metastases. Manuel is devastated and having difficulty with body image and preoccupied about his current romantic relationship. While he has had time to consider a future other than professional baseball, he grieves the loss of his arm and wonders if there is any point to continuing the treatment at all. He complains of acute pain but is hesitant about the proposed pain medications due to his awareness of addiction potential. Unfortunately, the pain keeps him awake at night, worsening his anxiety and feelings of helplessness and hopelessness. His parents are distressed because he is questioning his long-standing religious beliefs as a result of his advancing disease.

## Medical Approaches at the End-of-life

### Palliative Care

Palliative care is a cost-saving resource focused on optimizing QOL via symptom control and medical decision-making among those living with a serious illness. This resource care can be helpful at any stage of illness, ideally soon after a person is diagnosed [6, 7]. Patients receiving palliative care are not required to stop the active treatment designed to cure a serious illness. Palliative care is increasingly becoming the standard of practice in caring for those living with serious illnesses [7, 8], but access to this valuable service is limited in many countries [9]. Incorporation of palliative care approaches involves specific approaches to elicit goals of care from patients and their families, documentation of these goals in medical records, and planning clinical care and symptom control accordingly [10•].

### Hospice

Although hospice care is frequently used interchangeably with palliative care, and there is substantial overlap, it is not synonymous. Like palliative care, hospice programs provide continuous care aimed at reducing the burden of physical, psychological, and spiritual suffering for patients with life-limiting illnesses [11]. The emphasis of hospice care is not solely on symptom management but also on the promotion of personal values and cultural considerations to guide decision-making and provide comprehensive assessment of needs or preferences. Hospice providers also support bereaved families, with many programs including support for family members in the year after death, a service less common in hospital settings [12, 13]. Palliative care can transition to hospice care if an adult patient is likely to die within 6 months and efforts to cure the disease have ceased. For adolescents, the 6-month time requirement does not apply and teens can receive curative treatments concurrent with hospice services.

### End of Life in the Hospital

Since the 1990s, a majority of pediatric patients with severe and/or progressive chronic diseases have received much of their care at tertiary or quaternary healthcare centers [14, 15]. Prolonged admissions for end-of-life cases are neither cost-effective nor ideal patient care. In the USA, one barrier to transitioning patients to home care is financial. Health insurers do not always adequately reimburse for home palliative and hospice care, often pressuring parents to return to the hospital as medical care grows too complicated for families to manage. Hospital teams should strive to involve in-house palliative care teams as soon as possible as their inclusion is associated with fewer hospital days and a higher likelihood of death at home [10•]. Dying at home may be the right choice for some families. Adolescent and young adult patients are able to maintain a closer sense of normalcy with familiar surroundings, which promotes greater sense of control, autonomy, and privacy [16]. Other families may prefer the child *not* die at home for fear of the traumatic memory of death in the home, worries about lasting impacts on siblings, and doubt pertaining to the ability to effectively manage daily symptoms [16, 17].

## Symptom Management

Adolescents and young adults approaching the end of life are most likely to experience physical symptoms of fatigue (57–86%), decreased mobility (76%), pain (73%), poor appetite (71%), and dyspnea (6–21%) [18, 19]. In the last week of life, dyspnea and pain are most common [20]. The most common psychological symptoms in the last month of life include sadness or grief, anxiety, fear (of being alone, death, and/or pain), and guilt [20]. They may also feel isolated from peers [21•].

First-line approaches to effectively address troubling physical and psychological symptoms include behavioral and environmental interventions. At baseline, such interventions include honest, confidential, and culturally sensitive communication with health providers, input into plans for symptom relief, discussions about the level of assessment and medical interventions, access to beneficial coping skills, a predictable schedule, familiar activities, achievable life goals, and support from loved ones [22••, 23]. Specifically in patients aged 16–24, interventions may emphasize the role of independence and social support, as these are critical aspects during this developmental period.

More specific behavioral and environmental interventions can target identified concerns. For example, those who feel isolated may find comfort in social media groups and/or connections with terminally ill peers [21•]. Those with reduced mobility and fatigue might find the use of a walker or wheelchair helps conserve energy while maximizing independence [23]. Earplugs, eye masks, noise reduction, white noise machines, and lighting optimization may reduce environmental contributors to sleep disturbances [24]. Because loss of appetite may result from the body’s need to conserve energy and from its decreased ability to utilize food and fluids, patients should be allowed to choose if and when to eat or drink, and may prefer to eat much smaller amounts or foods that require less chewing. Relaxation and distraction techniques, as well as increasing choices and control, can be helpful for physical as well as psychological well-being.

Ideally, all patients approaching the end of life would have access to psychological support and targeted consultation from a mental health professional. Traditional psychotherapy approaches (cognitive behavioral therapy, acceptance and commitment therapy, and mindfulness-based cognitive therapy) and/or those targeted for end of life (dignity therapy, individual meaning-centered psychotherapy) are helpful evidence-based options. Cognitive behavioral therapy is associated with reduced fatigue levels, higher QOL, improved mood, less pain, and less distress among cancer patients [25]. Acceptance and commitment therapy has been shown, via meta-analysis, to significantly reduce cancer patients’ psychological distress, improve psychological flexibility, increase QOL, and increase one’s sense of hope [26•]. A recent systematic review concluded that mindfulness-based interventions led to statistically significant improvements in sleep disturbances, pain, anxiety, depression, and cancer-related fatigue among cancer patients [27•].

Dignity therapy (DT) is a manualized intervention developed for use in palliative care settings to enhance a sense of meaning and purpose for patients, help them identify their legacy, and preserve their dignity [28••]. In DT, patients are invited to discuss the aspects of their life they would most want remembered. Sessions are guided by a framework of questions that are provided in advance of the session, in order to give the patient time to reflect on the questions and prepare answers. The recorded interview is transcribed into a narrative document that is provided to the patient to revise as desired. The final document is provided to the patient, who may share it with loved ones, if they wish. Randomized clinical trials have provided support for the use of DT for anxiety reduction [28••].

Individual meaning-centered psychotherapy (IMCP) is a manualized psychotherapy with three goals: (1) to process personal issues and feelings about their illness, (2) to facilitate a greater understanding about what provides meaning in life, and (3) to assist patients in maintaining this sense of meaning in the face of illness progression [28••]. Randomized clinical trials have provided support for the effectiveness of IMCP in improving spiritual well-being and reducing psychological distress compared to supportive care.

Traditional medical approaches can also be helpful for symptom control. For example, palliative radiation, or radiotherapy, can offer relief from physical symptoms (such as pain, breathlessness, cough, swelling, ulceration, bleeding, neurological deficits, and decreased mobility) and thus improve QOL in patients with metastatic disease [29]. Dosage and scheduling depends on prognosis, status of metastases, and primary site of disease.

Medications are another option for most patients at the end of life. Decisions regarding the use of medications should be made with input from the patient, be congruent with the patient’s life goals, take into account life expectancy, and be regularly reviewed to ensure optimal QOL. For example, at end of life, concerns about addiction, over-sedation, and/or drug-drug interactions need to be weighed with patient comfort and other life goals. Patients and their families might have different views about these goals, making communication with the patient and family essential, including an opportunity for the patient to have private conversations with medical staff [22••]. For instance, a parent might want the patient awake as much as possible while the suffering patient might prefer to receive pain medications that cause them to sleep much of the time.

Even with no chance of cure, palliative chemotherapy can be used to optimize symptom control and maintain QOL in advanced cancer [30, 31•]. When used within the shared decision-making model, palliative chemotherapy can mitigate the need for emergency room visits [31•] as well as improve other domains of suffering such as poor appetite, tiredness, and poor QOL [32].

With regard to cancer pain, the analgesic ladder developed by the World Health Organization (WHO) is the medication standard of care, with suggestions related to augmentation to this approach [33]. Per the WHO analgesic ladder, nonsteroidal anti-inflammatory drugs (NSAIDs) or acetaminophen (with or without adjuvants) is recommended for mild pain. Weaker opioids (hydrocodone, codeine, tramadol) (with or without non-opioid analgesics, and with or without adjuvants) are recommended for moderate pain. Stronger opioids (morphine, methadone, fentanyl, oxycodone, buprenorphine, hydromorphone, etc.) (with or without non-opioid analgesics, and with or without adjuvants) are recommended for severe, persistent pain. For acute pain, the initial therapy should be the strongest analgesic based on pain intensity. For those with persistent pain, medication should be given on a regular schedule with breakthrough medication available as needed. Due to neural adaptation of opiates, patients with a history of opioid use may need higher doses of opioids than expected for pain control or the addition of adjuvant medication to achieve adequate analgesia [34, 35].

Adjuvants can include additional opiates, antidepressants (tricyclic antidepressants, serotonin-norepinephrine reuptake inhibitors, etc.), anticonvulsants (gabapentin and pregabalin), topical anesthetics (lidocaine), topical therapies (capsaicin), corticosteroids, bisphosphonates, and/or cannabinoids [33]. The International Association for the Study of Pain has suggested focusing on the type of pain and on the mechanism of action of the drugs used to treat it. Thus, inflammatory pain may first be treated with steroids or NSAIDs, and neuropathic pain may be first treated with antidepressants or anticonvulsants, as examples [33]. Ketamine, a phencyclidine derivative, has recently been demonstrated to be helpful in the treatment of cancer-related neuropathic pain [36]. Ketamine has also proven effective at reducing acute pain in opioid-tolerant patients [37]. Neurolytic blocks and intrathecal pumps have shown efficacy in managing pain symptoms when traditional therapies have been ineffective [38••].

Opiates are considered the first-line medication for end-of-life dyspnea [39]. Psychostimulants (modafinil and methylphenidate) are sometimes used and may be beneficial for end-of-life fatigue. Steroids are another viable option; however, neither class of medication has been extensively studied for this indication [40]. Blood transfusions can also be used to increase energy levels in those with low platelets [41].

Psychostimulants may also be used at the end of life in order to elevate mood, counter opioid-induced sedation, increase appetite, improve cognition, and potentiate the analgesic effect of opioids [42–45].

Psychotropic medications FDA approved for mood and anxiety symptoms (SSRIs, SNRIs, TCAs) may be used in this setting, recognizing that some of them require weeks before reaching full efficacy. Thus, faster acting agents (such as stimulants) might be required at the end of life. Ketamine can be used for depressive symptoms as well [46–49]. Not yet commercially available, psilocybin appears to have a positive impact on mood, anxiety and cancer-related psychiatric distress [50].

Benzodiazepines can be effectively used in patients with seizures, catatonia, air hunger, or severe treatment-resistant nausea, assuming there is no adverse reaction [51]. Despite little data supporting the use of benzodiazepines as an anxiety treatment, they are often prescribed for this indication as well. Some individuals dislike the effects and find it difficult to use coping skills while under their influence, others report paradoxical, agitated reactions. Additional concerns include increased risk for dependence, decreased slow wave (most restorative) sleep, anterograde amnesia, delirium, and worsening of PTSD, such as medical trauma [52–56]. Given these risks and lack of supporting data for their use for anxiety, benzodiazepines are not recommended for this indication unless other approaches have failed, patient prefers this medication, and/or patient is expected to survive only a few weeks or less [57••].

## Preparing Patients and Families for Death

Providing culturally sensitive care at the end of life requires the practitioner to keep in mind the resources, beliefs, experiences, and values of their patients and families [58••]. As examples, pain meaning and expression are impacted by cultural background; access to care and other comforts are driven by financial wealth; some families do not wish a terminal prognosis shared with the patient, others might feel an obligation to keep a clear mind free from mind-altering medications. While outside the scope of this brief paper, conducting a careful cultural assessment and developing an understanding of each unique patient and family have the potential to optimize the end-of-life experience [58••].

Young people planning for the end of life need to be supported in their choice of how to spend their last weeks or months [59]. Some may opt to exhaust all treatment options and advocate for aggressive treatments until the end. Others may opt to stop treatment all together and focus on palliative care or incorporate a blended approach. Regardless of the approach, patients and their families can focus on meaning and memory making. *Legacy building* is a term used to describe activities that allow patients to review their life and its meaning while creating a lasting memento that can be enjoyed by family and friends [60]. Psychosocial interventions that incorporate legacy building have been shown to improve parent–child communication at the end of life [61•]. Reviewing one’s life and its impact allows for the facilitation of difficult conversations and the expansion of family-wide understanding [62].

Coming to terms with one’s own death as an adolescent or young adult appears to differ from dying in more established adulthood. In legacy activities, young people appear to prefer future-oriented shorted term goals to the more traditional hindsight review of one’s life [63]. Patients in this developmental stage are also typically pursuing new occupational, educational, and interpersonal journeys that are quite suddenly and arbitrarily halted: first with a cancer diagnosis, and then with the realization of a cureless disease and shortened life [63]. Patients must not only grieve the future that they imagined but also face the distress of seeing peers continue the trajectories that they feel prohibited from pursuing (e.g., moving out and going away to college).

Multidisciplinary teams can work with patients to identify and accomplish goals. Foundations such as *Make a Wish* are often key in getting dream trips or events accomplished. For patients older than 18, foundations such as *Dream Foundation* or *Jack’s Helping Hand* can be contacted for similar bucket list arrangements. Oncology social workers are instrumental in this important rite of passage for young people with advanced cancer.

The acceptance of inevitable death in a teenager or young adult is a dynamic and devastatingly difficult process that takes time and differs for each member of the patient’s family. The increased incorporation of palliative treatments, as opposed to curative, entails a painful psychological shift from life preservation to life amelioration [64]. Besides the pursuit of meaning making activities, *advance care planning* is a critical step towards accepting and preparing for the death of a young person. Advance care planning encapsulates both legal decision-making plans and less formal proposals such as the creation and completion of bucket lists [64]. Patients nearing the end of life should be encouraged to complete an advanced directive (AD) that stipulates the medical care they accept or refuse (such as *Do Not Resuscitate* orders). Research shows that allowing adolescents and young adults to participate in the EOL decisional process helps parents and medical providers make educated treatment decisions, reduces emotional distress and regret, and may improve overall QOL by aligning medical decisions with the patient’s values and preferences [65]. In addition to completing an AD, non-legal guides such as Voicing My CHOiCES™ can help teens and young adults document both EOL preferences and wishes for eventual funeral and burial [66]. While the heart-wrenching nature of a young person’s death due to malignancy cannot be understated, the experience of dying is described as more peaceful when patients feel their choices are respected, they have made an impact on other’s lives, and they will be remembered [67].

Parents caring for a dying child also need to be supported. Qualitative research on the needs of parents caring for a dying adolescent or young adult reflects several important themes. Research on parents who have accepted the terminality of a cancer diagnosis reflects an important set of priorities. Parents need to have their child’s uniqueness recognized, to retain as many parenting duties as they can, to preserve as much normality in family life as possible, and to be heard and understood by their child’s medical team [68]. Professionals working with these parents can promote adjustment by helping parents identify ways to continue loving and caring for the patient as symptoms worsen, work through challenges both practical and emotional, and prioritize what matters the most to the family culturally and spiritually [69].

A multidisciplinary team-based approach is essential for communicating with families about the need to prepare for EOL. While oncologists may feel as though they have clearly informed patients of their impending death, studies show that only one in four families have prognostic expectations that match that of their oncologist’s [70]. Families have been shown to over rely on implicitly expressed optimism during physician encounters such as expressing hope or describing response to treatment rather than discussing the chance of long-term remission [70]. In a study of children who eventually died of cancer, physicians were aware of prognosis approximately 100 days before that of the patients’ parents [71]. The inclusion of mental health professionals in prognostic discussions can facilitate patient and family acceptance of and preparation for the impending death of their child [67].

## Recommendations and Conclusions

Returning to our case illustration, several steps can be taken to support Manuel through his understanding and acceptance of his prognosis. As outlined above, a multidisciplinary team meeting is one meaningful way to revisit goals of care and ensure patient and family understanding of available treatments and approaches. In joint collaboration, the psychologist and other palliative care team members could work to address sleep hygiene in an effort to minimize the impact of insomnia on Manuel’s QOL. Manuel could engage in individual meaning-centered psychotherapy in order to focus on meaning making in light of an abbreviated life and/or work with his social worker to achieve big items on his bucket list. For example, Manuel could reflect on his legacy as a baseball player, including the joy his family derived from seeing him on the field and teaching his younger brother how to play. Perhaps he could be gifted tickets to a professional baseball game from the *Dream Foundation* and have opportunity to meet his favorite player. The work should also include Manuel’s family, not only to recommend early engagement of home hospice but also to ensure that goals of care align with cultural, spiritual, and familial values. Inclusion of the team’s chaplain could assist Manual in his religious crisis and offer support for his grieving parents.

To summarize, there is no “one size fits all” for late adolescent and young adult patients at the end of life. The primary task of the multidisciplinary team is to understand and help implement the young person’s wishes in terms of medical treatments and palliation, help realize short-term goals, assist in the reflective process of legacy building, and ensure as much physiological comfort in the face of worsening disease. Team members should consistently strive to support grieving parents as they face the unimaginable task of bidding farewell to their child. The work may never feel complete, communication may never be perfect, but a considerate team will help the patient and family gain more peace by fostering honest and regular discussions that improve QOL.

## Data Availability

Not applicable, this is a review.

## Code Availability

Not applicable, this is a review.
